# Human liver myofibroblasts during development and diseases with a focus on portal (myo)fibroblasts

**DOI:** 10.3389/fphys.2015.00173

**Published:** 2015-06-23

**Authors:** Sébastien Lepreux, Alexis Desmoulière

**Affiliations:** ^1^Department of Pathology, University Hospital of BordeauxBordeaux, France; ^2^Department of Physiology, Faculty of Pharmacy, University of LimogesLimoges, France

**Keywords:** portal fibroblast, myofibroblast, hepatic stellate cell, alpha-smooth muscle actin, liver development, fibrosis, tumoral stroma

## Abstract

Myofibroblasts are stromal cells mainly involved in tissue repair. These cells present contractile properties and play a major role in extracellular matrix deposition and remodeling. In liver, myofibroblasts are found in two critical situations. First, during fetal liver development, especially in portal tracts, myofibroblasts surround vessels and bile ducts during their maturation. After complete development of the liver, myofibroblasts disappear and are replaced in portal tracts by portal fibroblasts. Second, during liver injury, myofibroblasts re-appear principally deriving from the activation of local stromal cells such as portal fibroblasts and hepatic stellate cells or can sometimes emerge by an epithelial-mesenchymal transition process. After acute injury, myofibroblasts play also a major role during liver regeneration. While myofibroblastic precursor cells are well known, the spectrum of activation and the fate of myofibroblasts during disease evolution are not fully understood. Some data are in accordance with a possible deactivation, at least partial, or a disappearance by apoptosis. Despite these shadows, liver is definitively a pertinent model showing that myofibroblasts are pivotal cells for extracellular matrix control during morphogenesis, repair and fibrous scarring.

## Introduction

In homeostatic state, myofibroblasts are absent from the normal adult liver. Myofibroblasts are stromal cells showing myoid features and involved in production or remodeling of the extracellular matrix (ECM) scaffold. Myofibroblasts are recruited from the transdifferentiation of local stromal cells. Historically, in the liver, it has been postulated that the hepatic stellate cell, also named Ito cell or lipocyte, was the provider of myofibroblasts: in this logic, myofibroblasts were called also transitional cells, activated stellate cells or myofibroblastic cells (French et al., 1988; Mak and Lieber, 1988; Bachem et al., 1989). But other studies have shown afterwards, that fibroblasts located in the connective tissue of the portal tracts are important providers of myofibroblasts (Tang et al., 1994; Tuchweber et al., 1996). This implication of portal fibroblasts as precursor cells of myofibroblasts was observed in human obstructive biliary diseases as well as in animal models (Desmoulière, 2007; Wells, 2014). Indeed, portal fibroblasts are involved as hepatic stellate cells in liver repair after injury and in tumoral reaction. Portal fibroblasts are also an important stromal cell playing a major role during the fetal liver morphogenesis.

## Myofibroblast definition

Since their first description in granulation tissue (Gabbiani et al., 1971), numerous studies have been published leading to remarkable progresses in the understanding of myofibroblast biological characteristics and of their participation in physiological and pathological situations (Hinz et al., 2012). Myofibroblasts exert traction forces by expressing α-smooth muscle (SM) actin and are able to participate in connective tissue remodeling by synthesizing ECM components, matrix metalloproteinases and their inhibitors. When the repair process is completed, in normal situations, myofibroblasts disappear by apoptosis (Desmoulière et al., 1995). Although presenting SM cell features, myofibroblasts however do not express h-caldesmon (150 kDa caldesmon) and smoothelin which seem to be specific for SM differentiation last step (Frid et al., 1992; van der Loop et al., 1997; Ceballos et al., 2000).

## Liver myofibroblast during liver morphogenesis

**Figure 1:**
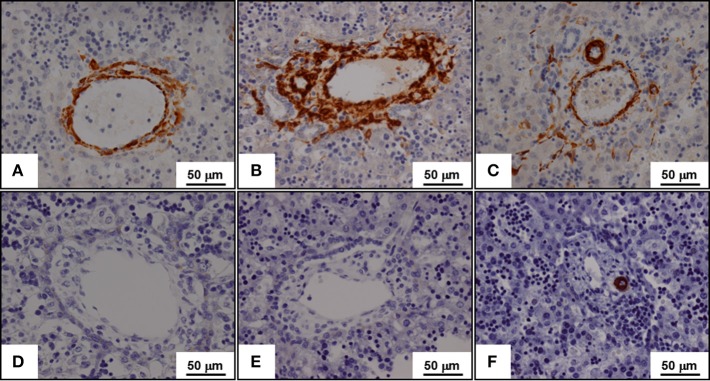
**Expression of α-smooth muscle actin (A,B,C) and of h-caldesmon (D,E,F) in fetal liver tissues during the lobulation of the fetal liver**. α-Smooth muscle (SM) actin is expressed by myofibroblasts while h-caldesmon is expressed by SM cells. The lobulation of the fetal liver begins near the liver hilum at the 9th week of development, and continues with a centrifugal pattern in the liver until about 1 month *post-partum*. Three stages of the portal tract maturation are described. At the ductal plate stage, the portal vein is surrounded by myofibroblasts that express α-SM actin **(A)**; SM cells expressing h-caldesmon are not yet present **(D)**. At the ductal plate remodeling stage, α-SM actin expressing myofibroblasts surround the biliary tubular structures from the ductal plate, which were incorporated in the portal stroma **(B)**; again, h-caldesmon is not still present **(E)**. At the remodeled stage, α-SM actin expressing myofibroblasts disappear; only arterial tunica media SM cells expressed both α-SM actin **(C)** and h-caldesmon **(F)**.

Liver mesenchyme deriving from the mesoblast of the septum transversum is invaded by the epithelial part of the entodermal hepatic diverticulum during the 4th week of development (WD) of normal human embryo (Roskams and Desmet, 2008). The lobulation of the fetal liver begins near the liver hilum at the 9th WD, and continues with a centrifugal pattern in the liver until about 1 month *post-partum*. Mesenchymal part of the liver gives birth to the sinusoid and perisinusoidal space in the future lobules and future portal tracts at the edge of the lobules, including finally portal fibroblasts (Asahina et al., 2011). Each mesenchymal compartment shows a specific maturation pattern (Figure 1).

-The portal tract maturation follows a sequence classically divided in three stages. At the first stage, *the ductal plate stage*, a mesenchymal future portal tract containing a large branch of portal vein and limited stroma is surrounded by segments of double-layered cylindrical or tubular structures. At the second stage, *the ductal plate remodeling stage*, the future portal tract incorporates the tubular structures into the stroma and branches of hepatic artery develop. At the last stage, *the remodeled stage*, the portal tract is mature and is characterized by a normal connective tissue containing a branch of portal vein, two branches of the hepatic artery and two bile ducts (Crawford et al., 1998).
-The mesenchyme of the septum transversum framework gives rise to sinusoidal compartment, which comprises endothelial and mesenchymal cells entrapped in the perisinusoidal space (future Disse's space). Endothelium of the sinusoid is continuous in the beginning of development and discontinuous after the 12th WD (Enzan et al., 1997). Perisinusoidal mesenchymal cells contain the developing hepatic stellate cells (Wake, 2006), which the embryonic origin is controversial (for review, see Geerts, 2004).

Other mesenchymal cells in the septum transversum can differentiate into fibroblasts, which occupy the subcapsular connective tissue of the liver (Enzan et al., 1997).

Stromal cells with myoid features, called myofibroblasts by Libbrecht et al. (2002), are specially implicated in the maturation of the future portal tract (Villeneuve et al., 2009). During the first stage, future portal tract stroma contains myofibroblasts, which surround also portal vein branch. At the second stage, myofibroblasts surround developing bile ducts, developing arterial branches and portal vein. Outside these areas, portal myofibroblasts give place to fibroblastic cells, which do not express α-SM actin. During the maturation of the arterial branches, the tunica media myofibroblasts are replaced by SM cells, which express h-caldesmon. At the last stage, myofibroblasts have disappeared from the portal tract. On these morphological data, we suggest as other a potential role of the portal myofibroblasts during the maturation of biliary tree (Libbrecht et al., 2002; Villeneuve et al., 2009).

Quite the opposite, myofibroblasts are poorly implicated in perisinusoidal maturation. Numerous cellular retinol-binding protein-1 positive hepatic stellate cells extend cytoplasmic processes from the 13th WD, but only few of them also express α-SM actin (Geerts, 2001; Villeneuve et al., 2009).

## Liver myofibroblast as a repair cell during adult liver injury response

Depending on the predominant tropism and the duration of the injury, different patterns of liver inflammation are described. First, two preferential tropisms of hepatitis can be separated in theory: hepatitis with lobular tropism such as viral hepatitis or hepatitis with portal tropism such as biliary obstruction diseases. On light microscopy, a liver zone was preferentially injured depending on the etiology but the other zone is often involved. Two, with the duration and the severity of the injury, the parenchyma architecture can become entirely modified.

In case of acute hepatitis, liver morphology shows inflammatory cell infiltration and hepatocellular damage. The regression is characterized by macrophage cleaning of the necrosis and regeneration. Gradually, these residual changes fade and with times, the liver architecture returns to normal (*restitutio ad integrum*). During this process, hepatic stellate cell play a major role (Kordes et al., 2014). In addition, after partial hepatectomy, incredible liver regeneration capacities are obvious and hepatic stellate cell-derived myofibroblasts can become progenitors, including epithelial progenitors, participating in this specific property of the liver (Swiderska-Syn et al., 2014).

In case of chronic hepatitis, the repetition and/or the persistence of the injury lead to extensive involvement of the inflammatory reaction in the organ. Then, a chronic scarring process destroys the normal architecture of the organ leading to fibrosis and finally cirrhosis. The scars are characterized by extensive fibrous septa due to accumulation of collagenous ECM. They surround regenerative nodules formed by hepatocyte hyperplasia. Profound disturbance of the liver vascular bed accompanying cirrhosis is characterized by venous thrombosis and anarchic angiogenesis within the fibrous scars, sinusoid remodeling and capillarization within the regenerative nodules (Bosch, 2007). Myofibroblasts are the producers of the ECM constituting the scars. But fibrosis is now not considered as a static state, because it can be modified in structure or remodeled in composition in regard to the extraordinary capacity of liver regeneration (Schuppan et al., 2001). Depending on the stimulus, myofibroblasts can contribute to fibrosis regression by releasing of ECM degrading proteases.

## Origin of the myofibroblast involved in adult liver repair process

Depending on the duration of the injury, activated stromal cells in acute inflammation and myofibroblasts in sub-acute/chronic inflammation are recruited as a reaction to the lesion.

### Local production

**Figure 2:**
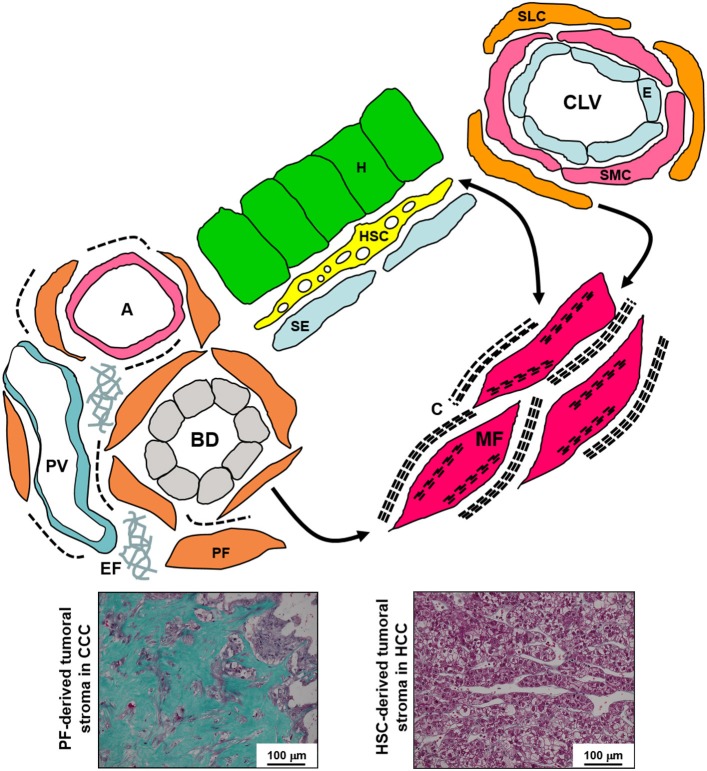
**Schematic diagram of the various liver fibroblastic cells able to acquire a myofibroblastic phenotype and involved in fibrogenesis and tumoral stroma formation**. The portal fibroblasts (PF) located in the portal tract connective tissue around bile ducts (BD), portal arteries (A), and portal veins (PV), and the second-layer cells (SLC), fibroblasts located around the smooth muscle cells (SMC) and the endothelium (E) of the centrolobular veins (CLV), can acquire a myofibroblastic phenotype, and these cells do not seem to be able to reacquire a quiescent phenotype; in contrast, the hepatic stellate cells (HSC) containing lipids droplets and located in the Disse's space between the hepatocytes (H) and the sinusoidal endothelium (SE) can modulate their myofibroblastic differentiation, and present pericyte-like features suggesting that they function as liver-specific pericytes participating in the regulation of sinusoidal blood pressure. Myofibroblasts (MF) present microfilament bundles and secrete large amounts of extracellular matrix. In addition, PF and PF-derived MF are the major, if not the only, cells that produce elastin. Numerous PF-derived MF are involved in the formation of the abundant fibrous stroma present in cholangiocarcinoma (CCC) while generally rare HSC-derived MF are present in the scanty tumoral stroma of hepatocellular carcinoma (HCC) (Masson's trichrome histochemistry). C, collagen; EF, elastic fibers.

Depending on the site of injury—portal, lobular or both—, the corresponding stromal cells can be activated into myofibroblasts (Figure 2).

#### Portal fibroblasts

Fibroblasts maintain the connective tissue architecture *via* the ECM that they secrete, but because they are a heterogeneous population of connective tissue cells, they have specific functions depending on their embryological origin and depending *in fine* on their organic site (Rinn et al., 2006, 2008; Tschumperlin, 2013). The fibroblasts located within the connective tissue of the portal tract—the portal fibroblasts—give rise to portal myofibroblasts, which are involved in portal fibrosis, notably in congenital biliary malformations and acquired biliary diseases in human (Ozaki et al., 2005) or after common bile duct ligation in animal models (Tuchweber et al., 1996; Kinnman et al., 2003). It is well known that hepatic stellate cells are also activated when the peripheral lobular parenchyma is invaded by the inflammatory reaction (Tuchweber et al., 1996; Kinnman and Housset, 2002). However, data concerning the origin of the myofibroblasts during portal fibrosis, i.e., hepatic stellate cells or portal fibroblasts, as well as the kinetic of this cellular contribution are controversial: in murine models, for Mederacke et al. (2013), hepatic stellate cells are the principal providers at a late time point of the injury, while it was not the case for Beaussier et al. (2007). Nevertheless, portal fibroblasts and myofibroblasts definitively have an important role in the biliary patterning. They participate in the polarity maintenance and the proliferation regulation of the cholangiocytes (Jhandier et al., 2005; He et al., 2008; Tanimizu et al., 2012). In the same way, interactions between myofibroblasts and biliary cells are also important in the ductular reaction and fibrosis development during the chronic bile duct diseases. In rat model of biliary fibrosis, reactive ductules express growth factors such as platelet-derived growth factor, connective tissue growth factor, or transforming growth factor-β2, which activate portal fibroblasts and increase matrix deposition (Milani et al., 1991; Grappone et al., 1999; Sedlaczek et al., 2001). Accompanying these epithelial-mesenchymal interactions, myofibroblasts produce tenascin and type IV collagen, which play an important role in biliary development and activation (Terada and Nakanuma, 1994; Lamireau et al., 1999). Portal fibroblasts and myofibroblasts could be also involved in portal vasculature and nerve development (for review, see Wells, 2014).

#### Hepatic stellate cells

Hepatic stellate cells, which account for about 5–8% of cells in the normal liver, are characterized by a perisinusoidal distribution in the Disse's space and long processes extending along and around sinusoids, between the hepatocyte plates (Lepreux et al., 2004). The close association of hepatic stellate cells with endothelial cells resembles that of pericytes in capillaries. However, in normal liver, the endothelium is discontinuous and presents multiple fenestrations without diaphragms, allowing the rapid transport of solutes to the subendothelial space. In the normal liver, a basal lamina-like structure separates the two cell types but there is no true basement membrane. Hepatic stellate cells secrete collagens but, contrary to portal fibroblasts, they seem to not produce elastin (Lorena et al., 2004; Perepelyuk et al., 2013) even if, at least *in vitro*, hepatic stellate cell-derived myofibroblasts secrete tropoelastin into the culture medium (Kanta et al., 2002). On activation, the hepatic stellate cells acquire a myofibroblastic phenotype, contributing to the excessive ECM deposition observed in the pathological conditions of fibrosis and cirrhosis. Capillarization of the sinusoids also occurs, with a continuous endothelium formed, and the presence of a true basal lamina. The experimental model of carbon tetrachloride (CCl_4_) treatment in rats has been extensively used to study the involvement of hepatic stellate cells in liver fibrogenesis (Sakaida, 2008). Following chronic injury induced by CCl_4_ treatment, a large number of myofibroblastic cells accumulate around centrolobular veins; septa containing myofibroblastic cells expressing α-SM actin then develop between centrolobular areas, and large amounts of ECM are deposited (Reeves and Friedman, 2002). Elastin and α-SM actin are co-localized in septa developing after CCl_4_ treatment, but activated α-SM actin-positive hepatic stellate cells in the parenchyma do not contain elastin. Thus, in the CCl_4_ model, the typical activated hepatic stellate cells containing α-SM actin seem to play little or no part in elastin deposition (Lorena et al., 2004). These observations suggest that different liver fibroblast subpopulations are involved in deposition of the different ECM components.

#### Others cells

Other quiescent fibrocompetent cells can be activated into myofibroblasts: vascular tunica media SM cells (Andrade et al., 1999), second layer cells around the centrilobular veins (Bhunchet and Wake, 1992), and capsular fibroblasts in the Glisson's capsule (Blanc et al., 2005). Recently, a process of mesothelial-to-mesenchymal transition has been mentioned as a novel source of myofibroblastic cells (for review, see Fausther et al., 2013).

#### Epithelial-mesenchymal transition (EMT)

EMT defines a process in which epithelial cells acquire mesenchymal features (Kalluri and Weinberg, 2009). EMT, as well the reverse process of mesenchymal-epithelial transition, occurs normally during the fetal development notably through Hedgehog and Notch signaling pathways. The exploration of Hedgehog signaling pathway in case of human or rat liver fibrosis secondary to biliary obstruction showed that cholangiocytes could undergo EMT (Omenetti et al., 2011). Choi and Diehl (2009) have suggested that some quiescent hepatic stellate cells are transitional cells which can differentiate into epithelial cells or myofibroblasts. But, particularly in this domain, the lack of specificity of lineage markers or tracers, the kinetic of their expression and the fact that the *in vitro* conditions do not reflect the *in vivo* situations, give rise to conflicting results (for review, see Xie and Diehl, 2013).

### Systemic contribution

Some studies, particularly in advanced stages of fibrosis and cirrhosis, have shown that myofibroblasts may originate from bone marrow. For example, in a mouse model of chronic alcohol consumption, bone marrow-derived cells contribute to the development of α-SM actin expressing cells (Fujimiya et al., 2009). However, the real contribution of bone marrow-derived fibrocytes as a source of myofibroblats during liver fibrosis and cirrhosis remains a question of debates (Kisseleva and Brenner, 2012).

## Myofibroblasts in tumoral stroma

Hepatocellular carcinomas (liver-cell carcinoma) have numerous etiologies, notably chronic B and C virus infection or chronic alcohol abuse and often, hepatocellular carcinomas arise in livers showing cirrhosis, which is in itself a precancerous condition. Bile-duct carcinomas (cholangiocarcinomas) are less common than hepatocellular carcinomas.

During liver regeneration, myofibroblasts are involved in regenerative response, but they are also implicated in the tumoral stroma development (Lemoinne et al., 2013). These myofibroblasts derive locally from hepatic stellate cells and/or portal fibroblasts (Figure 2).

However, the nature of the tumoral stroma is totally different in hepatocellular carcinoma and in cholangiocarcinoma.

In hepatocellular carcinoma, except in rare forms of scirrhous or fibrolamellar hepatocellular carcinoma, tumoral stroma is scanty. Often, the tumoral stroma is mixed with the fibrous stroma of the surrounding cirrhosis. Interestingly, the vessels surrounding the tumoral plates are not sinusoids, but continuous capillaries with a true basement membrane. In contrast, in cholangiocarcinoma, the tumoral cells are surrounded by an abundant fibrous stroma containing numerous myofibroblasts (Darby et al., 2011). This stroma is sclerous, sometimes with calcification, may be extensive, and submerges the scanty tumoral tubules.

We suggest that in hepatocellular carcinomas, mainly hepatic stellate cells and SM cells (Lepreux et al., 2013) are involved in the formation of the discrete tumoral stroma while, in cholangiocarcinoma, essentially portal fibroblasts are responsible for the large ECM deposition. Certainly, targeting cancer-associated myofibroblats could be the key for optimal treatment in future therapies and preventing or reversing the myofibroblast activation could inhibit or at least reduce tumor growth (Rizvi et al., 2014; Heindryckx and Gerwins, 2015).

## Activation spectrum of the stromal cells (from unactivated stromal cells to mature myofibroblasts) and reversibility of the myofibroblastic differentiation

The phenomenon of stromal cell activation is related to the transdifferentiation into myofibroblast. But, depending on the intensity and the chronicity of the stimulus, stromal cells can be activated at different degrees producing a spectrum from cells showing mixed features of quiescence and activation to cells presenting typical morphological and functional characteristics of myofibroblasts. It is particularly true for the hepatic stellate cells which can express overlapping features during the progression from the quiescent state with for example, the presence of vitamin A metabolism markers (intra-cytoplasmic lipid droplets, vitamin A autofluorescence, cellular retinol-binding protein-1 expression) to the fully activated state with for example, the overexpression of α-SM actin and the overproduction of ECM components (Ramadori, 1991; Gressner and Bachem, 1995; Hautekeete and Geerts, 1997; Lepreux et al., 2001, 2004). From this point of view, hepatic stellate cells present a more malleable phenotype compared with portal fibroblasts. Indeed, differences have been reported between these two fibrogenic cell populations, concerning the mechanisms underlying myofibroblastic differentiation, activation, and deactivation (Guyot et al., 2006). After isolation from healthy rat liver and culturing under the same conditions, both hepatic stellate cells and portal fibroblasts acquire a myofibroblast phenotype. Hepatic stellate cell-derived myofibroblasts display rounded and spread morphological characteristics with an enlarged cytoplasm and, more important, a poor survival after two to three passages. In contrast, portal fibroblasts-derived myofibroblasts have more elongated morphological characteristics and proliferate over multiple passages. *In vivo*, during liver diseases, hepatic stellate cell- and portal fibroblast-derived myofibroblasts present different fates. By using a model of cultured precision-cut liver slices, the behavior of the myofibroblast subpopulations during remodeling differs depending on the experimental model, the pathological situation, and the disease cause (Guyot et al., 2007, 2010). Hepatic stellate cell-derived myofibroblasts can lose α-SM actin expression without undergoing cell death, whereas in similar conditions, portal fibroblast-derived myofibroblasts die by apoptosis. When liver myofibroblasts are cultured on a basement membrane-like substrate (Matrigel), they loss α-SM actin expression, reacquire cytoplasmic lipid droplets, and thus revert, at least partly, to quiescence (Sohara et al., 2002). In the mouse model of CCl_4_ induced liver fibrosis, although some myofibroblasts die by apoptosis (Iredale et al., 1998), other myofibroblasts revert to an inactive phenotype during regression of fibrosis (Kisseleva et al., 2012). During the fetal liver portal tract maturation, a same phenomenon of replacement of the myofibroblastic cells by fibroblastic cells was observed (Villeneuve et al., 2009). These data are in accordance with a relative plasticity of the stromal cells; however, we suggest that this plasticity is well established in hepatic stellate cell-derived myofibroblasts, knowing in addition that hepatic stellate cells clearly present pericyte-like features (Costa et al., 2001), whereas portal fibroblast differentiation in myofibroblasts is more complete and less reversible. The question of the regulation of the stromal cell activation is important to consider therapeutic strategies. For example, the blockage of Hedgehog or Notch signaling pathways in rodent models of liver fibrosis leads to partial deactivation of activated hepatic stellate cells and inhibition of fibrotic process (Chen et al., 2012a,b; Xie et al., 2013).

## Conclusion

In the past, many studies have been performed using cells derived from explants of human liver parenchyma (Win et al., 1993). Initially, it was suggested that mainly activation of hepatic stellate cells contributes to this population. It is now assumed that these cells are rather representative of many if not all the fibrogenic cell populations present in the liver. It is also accepted that these different fibrogenic cells present different features and that their mechanisms of activation and deactivation are definitively not identical. However, to our knowledge, no reliable markers have been identified that allow unambiguous discrimination between these different cell populations and particularly, between hepatic stellate cell- and portal fibroblast-derived myofibroblasts. However, clearly, depending on the cause of the lesion (e.g., virus, alcohol) and then the primary location of the injury, the fibrogenic cells involved are different. Knowing that the deactivation mechanisms of these different cells are not similar, the question of the reversibility of the liver fibrosis/cirrhosis remains a burning issue. Certainly, portal fibroblasts are involved in many pathological situations and must be considered as a major fibrogenic cell population beside the hepatic stellate cells. Finally, the pivotal role of the portal (myo)fibroblasts in the fetal liver development, as well as in wound healing, including tumoral stroma which could be assimilated to an overhealing wound (Schäfer and Werner, 2008), notably through their interactions with the proliferative bile structures, would require more investigations in the way of liver regeneration application.

### Conflict of interest statement

The authors declare that the research was conducted in the absence of any commercial or financial relationships that could be construed as a potential conflict of interest.

## Abbreviations

SM: smooth muscle
ECM: extracellular matrix
WD: week of development
CCl_4_: carbon tetrachloride
EMT: epithelial-mesenchymal transition.
